# Subcellular trafficking and transcytosis efficacy of different receptor types for therapeutic antibody delivery at the blood‒brain barrier

**DOI:** 10.1186/s12987-023-00480-x

**Published:** 2023-11-06

**Authors:** Mikkel Roland Holst, Nienke Marije de Wit, Burak Ozgür, Andreas Brachner, Kathrine Hyldig, Antje Appelt-Menzel, Hannah Sleven, Zameel Cader, Helga Eveline de Vries, Winfried Neuhaus, Allan Jensen, Birger Brodin, Morten Schallburg Nielsen

**Affiliations:** 1 Department of Biomedicine, Aarhus University, Aarhus, Denmark; 2 Department of Molecular Cell Biology and Immunology, Amsterdam UMC location Vrije Universiteit Amsterdam, Amsterdam, The Netherlands; 3 Amsterdam Neuroscience, Amsterdam, The Netherlands; 4 Department of Pharmacy, University of Copenhagen, Copenhagen, Denmark; 5 Biotherapeutic Discovery, H. Lundbeck A/S, Valby, 2500 Copenhagen, Denmark; 6 AIT Austrian Institute of Technology GmbH, Competence Unit Molecular Diagnostics, Centre for Health and Bioresources, Vienna, Austria; 7 Chair Tissue Engineering and Regenerative Medicine (TERM), University Hospital Würzburg, Röntgenring 11, Würzburg, Germany; 8 Translational Center Regenerative Therapies (TLC-RT), Fraunhofer Institute for Silicate Research ISC, Röntgenring 12, Würzburg, Germany; 9 Translational Molecular Neuroscience Group, University of Oxford, Oxford, UK; 10 Department of Medicine, Faculty Medicine and Dentistry, Private Danube University, 3500 Krems, Austria

**Keywords:** Blood‒brain barrier, Receptor-mediated transcytosis, Transferrin receptor, Sortilin, CD133, Podocalyxin, Brain endothelial cells, Caco-2, Therapeutic antibodies, Transcytosis, Cargo receptor

## Abstract

Here, we report an experimental setup to benchmark different receptors for targeted therapeutic antibody delivery at the blood–brain barrier. We used brain capillary endothelial-like cells derived from induced pluripotent stem cells (hiPSC-BECs) as a model system and compared them to colon epithelial Caco-2 cells. This approach helped to identify favourable receptors for transport into the cell layer itself or for directing transport for transcytosis across the cell layer. The sorting receptors transferrin receptor and sortilin were shown to be efficient as antibody cargo receptors for intracellular delivery to the cell layer. In contrast, the cell surface receptors CD133 and podocalyxin were identified as static and inefficient receptors for delivering cargo antibodies. Similar to in vivo studies, the hiPSC-BECs maintained detectable transcytotic transport via transferrin receptor, while transcytosis was restricted using sortilin as a cargo receptor. Based on these findings, we propose the application of sortilin as a cargo receptor for delivering therapeutic antibodies into the brain microvascular endothelium.

**Graphical Abstract:**

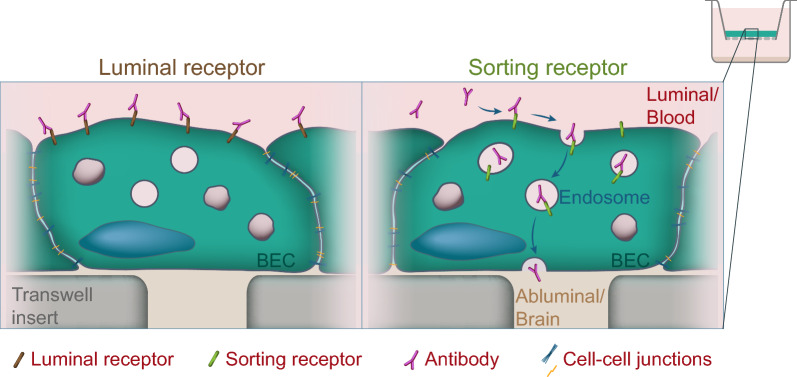

**Supplementary Information:**

The online version contains supplementary material available at 10.1186/s12987-023-00480-x.

## Highlights

- HiPSC-BECs can be used as a model system to study drug delivery via receptor-mediated transcytosis at the human blood‒brain barrier in vitro.
- The sorting receptors TfR and sortilin show significant internalization of cargo antibodies compared to the cell surface receptors CD133 and podocalyxin
- Sortilin is a putative cargo receptor for therapeutic delivery to brain endothelial cells

**Supplementary Information:**

The online version contains supplementary material available at 10.1186/s12987-023-00480-x.

## Introduction

Therapeutic antibodies can be delivered to the brain by using the transferrin receptor (TfR) as a cargo receptor for delivery across the blood‒brain barrier (BBB). This was demonstrated in rodents and nonhuman primates [44]. However, there is a need to use models of the human BBB for studying therapeutic delivery in humans and to limit the use of animal experimentation. To the current state-of-the-art human BBB models belongs a test system generated from human induced pluripotent stem cells differentiated into brain capillary endothelial-like cells (hiPSC-BECs) [19]. The resulting cell phenotype is subject to some controversy [19, 20] regarding the resemblance to human primary brain capillary endothelial cells. While considering this notion, there are several useful properties of the hiPSC-BEC-based BBB model, such as tight barrier properties and maintained functionality of major endothelial transporter proteins and receptors [6], which makes the BBB model suited for exploratory therapeutic research.

Genentech provided proof-of-concept for using cargo receptors for delivering therapeutic antibody constructs. They designed bispecific antibodies with TfR specificity on one arm and specificity towards the β-secretase enzyme BACE [40] on the other arm. This strategy proved significant for therapeutic delivery to the brain through the BBB via TfR-mediated transport [44]. Furthermore, it was established that the affinity of binding between the antibody arm targeting the cargo receptor (TfR) is important to enable transcytosis to the brain parenchyma [2, 45]. Many studies have focused on TfR as a cargo receptor, and recently, Basigin [5, 49], CD98hc [49] and many others have been proposed [47]. With different functional requirements of the vast amount of receptors expressed in the cell, it is important to consider whether receptors are available for cell surface binding and internalization of cargo antibodies and, importantly, whether the function of the cargo receptor is still maintained [2]. Previously, the Stanimirovic group addressed an important property in drug delivery: endosomal sorting of cargo receptors plays a pivotal role in the effective transcytosis of cargo antibodies [9]. Using an immortalized rat endothelial cell line, the researchers found that cargo antibodies sorted through the EEA1-marked sorting compartment are subject to transcytosis, whereas cargo antibodies, which continue into the late endosomal compartment marked by Rab7, are prone to end in the brain endothelial cell lysosomal degradative compartment [9]. Recently, another twist was added to the brain endothelial cell (BEC) sorting compartment when it was found that Rab7 could also play an active role in carrier formation for regulating polarized transcytosis of α-synuclein from the luminal to abluminal cell surfaces [1]. These findings suggest that different cargo formats are handled differently in the BEC sorting compartment. Thus, it is crucial to characterize the sorting properties of putative therapeutic cargoes within each format.

Benchmarking of different receptors for their functionality as antibody cargo receptors is an important aspect in drug delivery, as different receptors might prove favorable for different therapeutic goals. This is relevant to consider because drug targets can be located in the BBB itself [17, 46] as well as cells within the brain [16]. Therefore, choosing a BEC receptor that favors intracellular accumulation in the BEC cell layer could serve as the best strategy to alleviate BEC-related dysfunction [46]. On the other hand, for delivery to impact brain neurons, a BEC receptor favoring cargo transcytosis through the BBB towards the brain parenchyma is the best strategy.

In this study, we investigated two types of receptors for their use as cargo receptors in human BECs. We chose the two sorting receptors TfR and sortilin, which maintain recycling of transferrin and retrograde sorting of a diverse set of ligands, respectively. We also selected the two cell surface receptors CD133 and podocalyxin, localized in the apical (blood-facing) membrane and thus available for cargo antibody binding. We found that sorting receptors are effective for the internalization of antibody cargoes, whereas polarized cell surface receptors prove static and inefficient for the internalization of cargo antibodies. The experiments were compared using Caco-2- and hiPSC-BEC-based Transwell models as test systems. Our comparative Transwell setup with Caco-2 cells and hiPSC-BECs recapitulated the previously documented in vivo transcytosis functionality of a bispecific TfR-directed antibody construct [44] in hiPSC-BECs but not in Caco-2 cells. We further identified sortilin as an efficient cargo receptor for the internalization of therapeutic antibodies into the hiPSC-BEC layer.

## Materials and methods

All plastic ware was obtained from Corning or Greiner BIO-ONE unless otherwise stated.

### Antibodies

Monoclonal antibody sequences for this study were designed with the purpose of expressing both bivalent and monovalent molecules using controlled fab-arm exchange (cFAE, see Additional file 2: Fig S1E) developed and published by Genmab [15]. In short, mutations of either K409R or F405L were introduced into the control mab (here annotated B12) and cargo mab sequences, respectively.

Monoclonal antibodies were expressed using transient transfection of HEK293 6E cells. A proprietary vector containing the protein sequences for both antibody light and heavy chains was transfected into the cells using linear PEI as the transfection reagent. After approximately 7 days of antibody secretion, the cell medium was harvested by centrifugation and filtration. Antibodies were purified in a three-step process by protein-G Sepharose (GE Healthcare) affinity chromatography followed by desalting into PBS. The purified antibodies were further purified using ion-exchange chromatography (IEX) followed by size exclusion chromatography (SEC) according to standard protocols.

The duobody reaction between the mAbs of interest was facilitated using 1 M 2-MEA (Thermo Fisher, cat. no.: 156-57-0). The reaction was incubated at 31 degrees for 5 h followed by desalting into PBS using a Zeba Spin Desalting column (Thermo Fisher, cat. no.: 89893).

#### Cargo receptor antibodies

Prior to the analysis, all antibody specificities were verified using western blotting as well as immunocytochemistry testing of both human and mouse protein specificity (see Additional file 2: Fig S1 for example).

### Cell lines

Human induced pluripotent stem cells (hiPSCs) named SBAD-02-01 were obtained from IMI StemBANCC and tested for properties resembling the IMR-90 cell line used to obtain brain endothelial characteristics [18]. Caco-2 cells were obtained from ATCC and used from passage 5. Commercially available human brain progenitor-derived astrocytes were obtained from Gibco (cat. No.: N7805-100) and Lonza (cat. No.: CC-2565).

### Maintenance of cells

All cell lines were cultivated at 37 °C with 5% CO_2_, 95% air and saturated humidity.

SBAD-02–01 cells were maintained on Matrigel (BD Biosciences, cat. No.: 354230)-coated plates with mTeSR + medium (STEMCELL^™^ Technologies, cat. No.: 85850), as previously described [34].

Astrocytes were maintained on Matrigel-coated plates in astrocyte medium containing Dulbecco’s modified Eagle’s medium (DMEM, Gibco, cat. No.: 31966–021) supplemented with 1% N-2 supplement (Gibco, cat. No.: 17502–048), 10% fetal bovine serum (FBS, Sigma‒Aldrich, cat. No.: F9665), and 20 ng/mL human recombinant epidermal growth factor (EGF, Gibco cat. No.: PHG0314), with media renewed every third day.

Caco-2 cells were maintained on plastic ware using DMEM (Gibco, cat. No.: 31966021) with penicillin/streptomycin 10.000 U/Ml–10 mg/mL, 1% nonessential amino acids (Gibco, cat. No.: 11140050) and 10% FBS.

### Differentiation of hiPSC SBAD-02–01 into BECs

**Diagram. 1:**
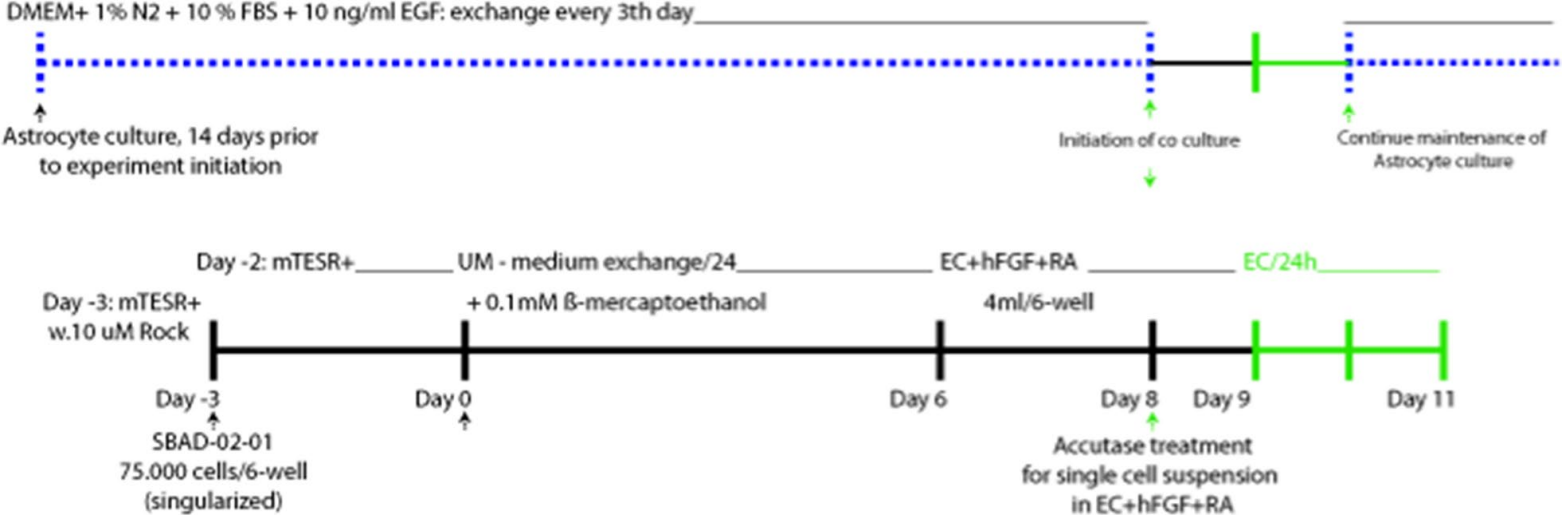
Timeline for hiPSC-BEC differentiation and noncontact coculture with astrocytes

We used a previously described protocol [34] with minor modifications (see Diagram 1). Three days before initiating BEC differentiation (D-3) and a minimum of four passages after thawing SBAD-02-01 cells, the cells were washed with Dulbecco′s phosphate-buffered saline (DPBS), dissociated with Accutase (Gibco, cat. No.: A1110501) for 8 min and resuspended in single-cell solution. Cells were then collected by centrifugation (300 × *g* for 5 min), and 75,000 cells were plated onto Matrigel-coated 6-well plates in mTeSR1 + medium with 10 µM ROCK inhibitor Y-27632 (Tocris Bioscience, cat. No.: 1254). Cells were cultivated in mTeSR + for 3 days (medium exchange on D-2) before BEC differentiation was initiated. On day D0, the medium was switched to unconditioned medium (UM) containing DMEM/F12 without L-glutamine (Gibco, cat. No.: 21331020), 20% (v/v) KnockOut^™^-Serum Replacement (Gibco, cat. No.: 10828028), 1% (v/v) MEM nonessential amino acid solution (Gibco, cat. No.: 11140050), and 0.5% (v/v) GlutaMAX™ (Gibco, cat. No.: 35050061). UM was replenished, and 0.1 mM β-mercaptoethanol (Sigma‒Aldrich Cat. No.: M3148) was added freshly every day (1:143.000 dilution of 14.3 M stock) for six consecutive days. On day six (D6), the UM medium was switched to EC medium consisting of human endothelial serum-free medium (Gibco, cat. No.: 11111044) supplemented with 0.5% B-27^™^ supplement (50X) (Gibco, cat. No.: 17504001), 20 ng/mL hFGF (R&D Systems Cat. No.: 233-FB) and 10 µM retinoic acid (RA) (Sigma‒Aldrich R2625) (EC medium). On day eight (D8), the cells were washed with DPBS, dissociated with Accutase for 15 min at 37 °C, and resuspended in single-cell solution. Then, 500,000 cells were seeded in EC medium onto collagen IV/fibronectin-coated Transwell thinserts (12-well format, 1.13 cm^2^, GREINER BIO-ONE, Cat. No.: GR-665641) with or without astrocyte noncontact coculture. On day nine (D9), the cell medium was exchanged with EC medium without hFGF and RA. Experimental culture was initiated on day 10 (D10).

### Experimental hiPSC-BEC culture

Cell monolayers were cultured on permeable supports in a 12-well Transwell setup using Thincerts cell culture inserts (GREINER BIO-ONE, Cat. No.: GR-665641). HiPSC-BEC culture: Astrocytes were seeded for coculture three weeks prior to the experiment at 10,000 cells per well in a 12-well plate and maintained in astrocyte medium until the initiation of coculture. SBAD-02-01 cells were predifferentiated into hiPSC-BECs, and at D8, 500,000 cells were seeded per 12-well filter insert. The filter insert with hiPSC-BECs was placed over astrocyte culture plates for 2 days (D8-D10, see diagram 1). On the experimental day, hiPSC-BECs were removed from astrocytes and placed in a new 12-well plate with experimental medium prewarmed to 37 °C. EC medium (without hFGF and RA) was used for mouse cargo antibodies, and endothelial cell growth medium 2 (ECM-2, PromoCell, cat. no.: C-22211) was used for human cargo antibodies due to interference between EC medium and the ELISA.

### Experimental caco-2 culture

Caco-2 cells were thawed and seeded directly onto 12-well Transwell inserts and grown to full confluency on the inserts (10 days) using DMEM (Gibco, cat. No.: 31966021) with penicillin/streptomycin 10.000 U/mL–10 mg/mL, 1% nonessential amino acids (Gibco, cat. No.: 11140050) and 10% FBS (Sigma‒Aldrich, cat. No.: F9665). Then, the cells were differentiated for 17 days using the same medium without FBS (SF-DMEM) prior to experimentation with medium exchange every third day. All cargo antibody experiments were performed in SF-DMEM.

### Transport experiment

The medium was exchanged on the experimental day to a 1200 µl bottom chamber and 475 µl top chamber. Cells were then left to recover from the medium change for 3 h before adding cargo antibodies and starting the transport experiment. The medium type was chosen dependent on the species of cargo antibody to avoid interference with the capture antibody of the ELISA (see section Experimental hiPSC-BEC culture for medium type). The transport experiment was started by spiking the media in the top chamber directly with 25 µl of 200 µg/ml spike solution into 475 µl, giving a final concentration of 10 µg/ml cargo antibody.

### Western blotting

Cells were lysed in lysis buffer containing 1% Triton X-100, 150 mM NaCl, 2 mM MgCl_2_, 2 mM CaCl_2_, and complete mini protease inhibitor cocktail (Roche), and 20 mM DTT was added (samples for immunoblotting with mouse anti-podocalyxin (R&D Systems MAB1658) were not treated with DTT). NuPAGE LDS sample buffer (Invitrogen, NP0007) was added to the samples, which were heated to 95 °C for 5 min. Samples were loaded into 4–12% bis–tris gels next to SeeBlue™ Plus2 prestained protein standard as a protein marker in a running buffer prepared from 20 × NuPAGE MES buffer, ddH2O, and NuPAGE antioxidant. Samples were run for 45 min at 140 V. Gels were then transferred for blotting using a Novex iBlot dry blotting system (Invitrogen, cat# IB24001) and blocked in Tris-buffered saline (TBS) with 0.1% Tween-20 and 5% milk. The blots were incubated with primary antibodies at 4 °C overnight and washed in 0.1% Tween-20 in PBS, followed by incubation with secondary antibodies for 1 h at RT. After washing in 0.1% Tween-20 in PBS, the blots were developed using ECL substrate (Pierce, 32106) and detected using an iBright 1500 (Invitrogen) chemiluminescence imager.

### Immunofluorescence and confocal microscopy

Samples were fixed with cytoskeleton fixation buffer containing 10 mM MES, 3 mM MgCl_2_, 138 mM KCl, 2 mM EGTA, 0.32 M sucrose and 4% PFA for 20 min at room temperature. Cells were stained for colocalization analysis using conventional methodology, including 10 min of 0.1% Triton X-100 for permeabilization and 30 min of 2% BSA treatment for blocking, all in phosphate buffered saline solution (PBS). Primary antibodies were diluted as listed in Table 1 in blocking solution and incubated with the samples for 1 h at RT. Samples were then incubated with secondary antibodies at 1:200 dilutions for 30 min at RT. Cell nuclei were stained for 30 min with 0.6 µg/mL Hoechst 32,528 (Invitrogen H3569). The samples were mounted on glass slides using Dako fluorescence mounting medium (Dako, Glostrup, Denmark S3023). Confocal images were captured by an Olympus IX-83 fluorescence microscope with a Yokogawa CSU-X1 confocal spinning unit and a Hamamatsu Orca Fusion BT C15440 camera, Olympus UPLSAPO W, × 60/1.20 NA water objective lens, using Olympus CellSens software (Olympus). For each channel, the laser power was adjusted and applied independently.

**Table 1:** List of study antibodies with functionality and origin

Primary antibody	Usage of antibody	Dilution	Species	Source
Anti-Sortilin *(cargo receptor antibody)*	Transport experiment Immunocytochemistry Immunohistochemistry Western blotting	10 µg/ml 10 µg/ml 8 µg/ml 1 µg/ml	Mouse	In-house developed
Anti-CD133 *(cargo receptor antibody)*	Transport experiment Immunocytochemistry Immunohistochemistry Western blotting	10 µg/ml 10 µg/ml 25 µg/ml 1 µg/ml	Mouse	DSHB clone HB#7
Anti-Podocalyxin *(cargo receptor antibody)*	Transport experiment Immunocytochemistry Immunohistochemistry Western blotting	10 µg/ml 10 µg/ml 1.25 µg/ml 1 µg/ml	Mouse	R&D Systems MAB1658
Anti-B12/TfR *(cargo receptor antibody)*	Transport experiment Immunocytochemistry Western blotting	10 µg/ml 10 µg/ml 1 µg/ml	Humanized mouse antibody	In-house developed from Genentech [45]
Anti-B12/Sortilin *(cargo receptor antibody)*	Transport experiment Immunocytochemistry Western blotting	10 µg/ml 10 µg/ml 0.8 µg/ml	Humanized mouse antibody	In-house developed from Genentech [45]
Anti-B12/TfR-647	Immunohistochemistry	27 µg/ml	Humanized mouse antibody	In-house developed from Genentech [45]
Anti-TNP (Trinitrophenol) Control antibody	Transport experiment Immunocytochemistry	10 µg/ml 10 µg/ml	Humanized mouse antibody	In-house developed
Anti-EEA1	Immunocytochemistry	13 µg/ml	Rabbit	Abcam ab109110
Anti-Rab7	Immunocytochemistry	5 µg/ml	Rabbit	Abcam ab137029
Anti-TGN46	Immunocytochemistry	2.5 µg/ml	Sheep	AbD-Serotec AHP500GT
Anti-Rab8	Immunocytochemistry	4.4 µg/ml	Rabbit	Cell signaling CST-6975 T
Anti-ZO1	Immunocytochemistry	6.5 µg/ml	Rabbit	Lundbeck, Denmark
Anti-Human Fc	ELISA	500 ng/ml	Goat	Sigma I2136
Anti-Mouse Fc	ELISA	500 ng/ml	Goat	Sigma M2650
Anti-HRP-Human Fc	ELISA	1 µg/ml	Goat	Sigma A0170
Anti-HRP-Mouse Fc	ELISA	1 µg/ml	Goat	Sigma A0168
Anti-Mouse Alexa488	Immunocytochemistry	10 µg/ml	Donkey	Invitrogen A21202
Anti-Human Alexa488	Immunocytochemistry	10 µg/ml	Donkey	Biotium 13C0308
Anti-Sheep Alexa568	Immunocytochemistry	10 µg/ml	Donkey	Molecular probes A21099
Anti-Rabbit StarRed	Immunocytochemistry	5 µg/ml	Goat	Abberior STRED-1002

### Image and colocalization analysis

A total of 1800 images were generated and processed manually for this study using IMARIS software v8.2 (Bitplane). The co-occurrence between receptors or cargo antibodies and intracellular sorting markers was determined using IMARIS (Bitplane) software spot segmentation with a colocalization analysis threshold set to 0.5. Numbers were collected from 100 cells per condition in three independent experiments. The colocalization scores were calculated as described previously [11]:

$$Colocalization \,\,score=\left(\frac{\mathrm{total\,\, receptor\,\, or\,\, cargo\,\, antibody\,\, spots\,\, co}-\mathrm{occurring\,\, with\,\, marker\,\, spots\,}}{\mathrm{total\,\, receptor\,\, or\,\, cargo\,\, antibody\,\, spots\,}}\right)*100 \%$$

### Measurement of transcytosis

Transcytosed cargo antibodies were measured using Capture ELISA. In short, Nunc Maxisorp 96-well plates were coated with 500 ng/ml species- and FC region-specific antibodies (see Table 1) overnight at 4 °C in PBS. Plates were washed once and blocked with 2% BSA in PBS including 0.05% Tween 20 (PBST). Spike medium solution was used to generate concentration curves and to calculate the amount of cargo antibody transcytosed from the acceptor (bottom chamber). Plates were washed with PBST four times and incubated with cell media collected from the transport experiment for one hour at RT under rotation. Due to the components of the EC cell media types, we found that ECM-2 was compatible with the ELISA capture procedure for detecting human cargo antibodies and EC medium for detecting mouse cargo antibodies. Plates were washed four times with PBST and incubated with HRP-conjugated detection antibodies targeting the species-specific FC region for one hour at RT under rotation. Plates were washed with PBST four times, 100 µl TMB PLUS2 (Kementec 4395 L) substrate was added for detection, and the reaction was stopped using 100 µl 0.5 M sulfuric acid. The signal was measured at 450 nm absorbance using a plate reader.

### Postmortem human brain tissue

Postmortem human brain tissue was obtained from the Netherlands Brain Bank (NBB), Netherlands Institute for Neuroscience, Amsterdam. For this study, we selected the superior occipital gyrus of seven AD patients and six age-matched nondemented controls. The staging of AD pathology was evaluated according to the revised criteria of Braak and Braak [3, 4]. All material was collected from donors after written informed consent for brain autopsy and use of brain tissue and clinical information for research purposes. Age, sex, postmortem delay (PMD), Braak score, and cause of death of all cases used in this study are listed in Table 2.

**Table 2:** Patient details

Patient	Gender	Age	Braak	PMD (h)	Cause of death
Alzheimer’s disease 1	F	82	6	05:00	Dehydration, cachexia
Alzheimer’s disease 2	F	90	5	03:50	Dehydration
Alzheimer’s disease 3	F	87	5	06:55	Heart failure
Alzheimer’s disease 4	F	83	5	04:00	Dehydration
Alzheimer’s disease 5	M	77	5	05:39	Pneumonia
Alzheimer’s disease 6	F	70	6	04:00	Sepsis (possibly pneumosepsis)
Alzheimer’s disease 7	F	69	6	06:10	Urosepsis
Nondemented control 1	M	79	2	07:15	Myocardial infarction
Nondemented control 2	M	86	2	05:30	Cancer of urinary tract, cachexia
Nondemented control 3	F	77	1	05:40	Apnea. Starvation/dehydration
Nondemented control 4	F	75	1	05:30	Myocardial infarct
Nondemented control 5	M	73	2	08:00	Invasive fungal infection and bacterial pneumonia
Nondemented control 6	M	84	1	05:35	Heart failure

### Immunohistochemistry and quantification

For capillary detection, 5 μm cryosections were mounted on coated glass slides (Menzel Gläser Superfrost PLUS, Thermo Scientific, Braunschweig Germany), air-dried, and fixed in acetone for 10 min. Sections were subsequently incubated for 30 min containing 10% normal goat serum and incubated overnight at 4 °C with primary antibodies as indicated in Table 1. All antibodies were diluted in PBS supplemented with 1% bovine serum albumin (BSA; Roche Diagnostics GmbH, Mannheim, Germany). Alexa 488-labelled goat anti-mouse antibody was used to detect CD133, PODXL, and Sortilin. Alexa 555-labelled streptavidin or Alexa 488-labelled streptavidin (dilution 1:400, Life Technologies) was used to detect ULEX (Vector labs B-1065). Sections were incubated for one hour with their specific secondary antibody. Finally, sections were stained with Hoechst (dilution 1:1000, Molecular Probes) to visualize cellular nuclei and mounted with Mowiol mounting medium. Representative images were taken using a Leica DM6000 ip fluorescence microscope (Leica DM6000, Mannheim, Germany) with a 63X oil objective.

Quantitative analysis of the immunohistochemical levels of CD133, PODXL, Sortilin and TfR was performed on the grey matter of the occipital cortex of control and AD cases. For each case, 4 pictures spanning all cortical layers of the grey matter of the superior occipital gyrus were taken. The immunofluorescence intensity of the double fluorescence staining was quantified using ImageJ version 1.49.

### Statistics

Statistical tests were performed using Prism (v.9.5) (GraphPad Software). All data sets are based on three or more independent experiments. Bar plots present mean values with standard deviation error bars (SD). Different means were analysed using the tests indicated in the figure legends with *p < 0.05, **p < 0.01, ***p < 0.001, ****p < 0.0001 and nonsignificant (ns).

## Results

### Putative cargo receptors expressed in human brain capillaries

**Fig. 1:**
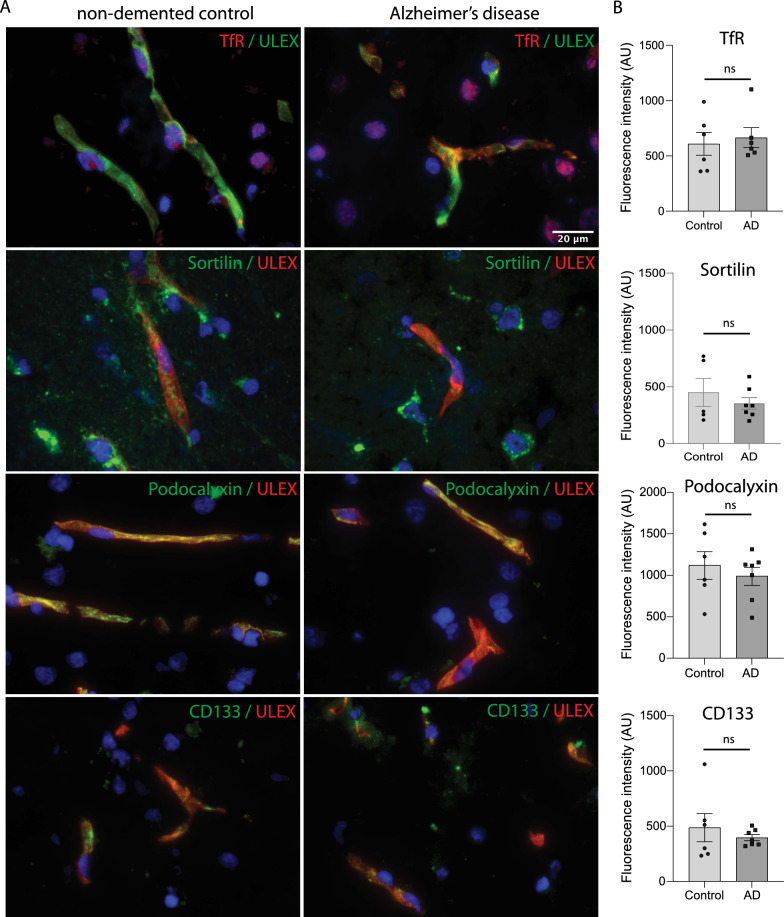
Sorting and luminal cell surface receptors are expressed in human brain microvasculature. **A** Micrographs showing immunohistochemical staining of tissue sections spanning all cortical layers of the grey matter of the superior occipital gyrus from human control (samples from six patients) and Alzheimer’s disease cases (samples from seven patients) for the indicated receptors. **B** Bar plots show quantification of the immunofluorescent signal within the endothelial vascular marker ULEX. Four areas were imaged and used for quantification from five control cases and seven AD samples per receptor. Statistical analysis was performed using Student’s unpaired t test. See also Additional file 2: Fig S1

The use of cargo receptors for antibody delivery depends on the expression of receptors in the BMEC layer in disease and health. To ensure that putative cargo receptors were present in human brain capillaries, we investigated the expression of the four chosen receptors (TfR, Sortilin, CD133 and podocalyxin) via immunohistochemistry on postmortem human brain tissue. The receptor staining was investigated in cortical layers of the grey matter of the superior occipital gyrus together with an endothelial vascular marker ULEX to image and quantify receptor expression in brain capillaries (Fig. 1A). We selected brain tissue from nondemented controls and Alzheimer’s disease patients (listed in Table 2) to test whether the putative cargo receptors were expressed in human brain capillaries and whether the expression level was affected in the case of Alzheimer’s disease (Fig. 1A). We quantified the signal intensity of the receptors within the vascular endothelial marker signal and found no difference in the expression of receptors between nondemented controls and Alzheimer’s disease cases (Fig. 1B). Sortilin has previously been shown to be highly enriched in various neuronal cells [33, 42], which could be the reason why areas outside of the endothelial marker show abundant expression of Sortilin (Fig. 1A). These results and previously published data [33, 43] confirmed the expression of the four receptors in the human BBB and their applicability as putative cargo antibody receptors for drug delivery into the brain.

### Verification of the transwell barrier model setup and putative cargo receptor expression

To study the transport of the selected cargo antibodies, we used a Transwell setup with cells seeded on the top chamber filter insert to form a barrier towards the bottom chamber, enabling analysis of intracellular sorting and cargo antibody transcytosis. HiPSC-BECs and the human epithelial cell line Caco-2 were chosen based on their excellent properties to model human cell barriers [10]. For the generation of hiPSC-BECs, we used a human skin-derived induced pluripotent stem cell line named SBAD-02-01 [32], which has no restrictions for commercial research. With this setup, we established a cell model with tight junction localization of ZO1 and an average TEER value of 1889 Ω cm^2^ (Additional file 2: Fig S2A, D). We used astrocyte noncontact coculture during the last 2 days of differentiation into BECs since we previously found astrocytes to affect the BEC sorting compartment [37]. High-throughput qPCR analysis using the Fluidigm^®^ Biomark platform^™^ on ten categories of markers confirmed the mRNA expression of endothelial markers such as *CLDN5* (claudin-5), *SLC2A1* (Glut1), *OCLN* (occluding) or *CDH5* (VE-cadherin) (also confirmed by immunofluorescence in Additional file 2: Fig S2F, see also Additional file 1: Table S1). Also, corresponding to the high Trans endothelial electrical resistance, the hiPSC-BEC cell layer also showed low paracellular permeability (p_Cells_ = 1,317 × 10^–7^ cm/s) of the small molecule sodium fluorescein (Additional file 2: Fig S2E). Caco-2 cells were differentiated using a previously described protocol [30] with a 17 day serum depletion on Transwell filters, resulting in tight junction localized ZO1 and an average TEER value of 600 Ω cm^2^ (Additional file 2: Fig. S2C, F). These two barrier setups were used throughout this study to investigate how cargo antibody trafficking and transcytosis can be exploited via different receptors in epithelial and brain endothelial-like cell layers.

**Fig. 2:**
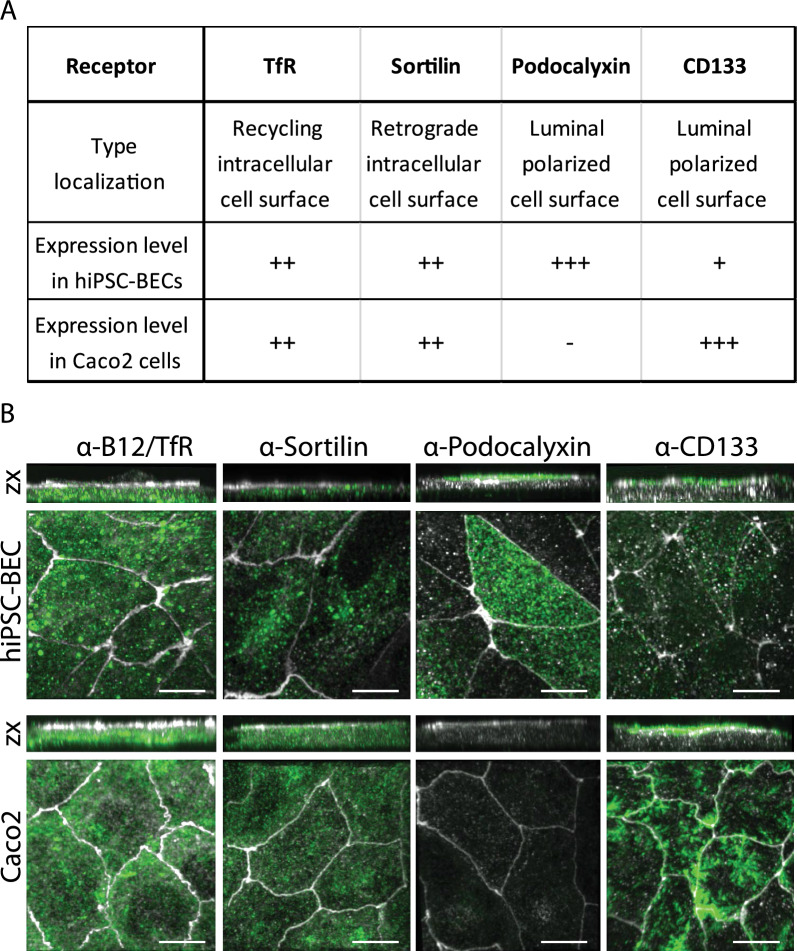
Characterization of receptor expression in two human cell barrier models. **A** Sorting receptors TfR and sortilin are both expressed in hiPSC-BECs and Caco-2 cells and localized at intracellular surfaces. The polarized receptors podocalyxin and CD133 are both expressed in the apical surface facing the luminal side of the Transwell chamber. **B** Micrographs showing maximum projected z-stacks with side view zx images above and xy images below. Cells are stained for ZO1 in white and the indicated receptors in green. Scale bars are 10 µm. See also Additional file 2: Fig S2

The recycling transferrin receptor (TfR) is well known for its functionality as a cargo receptor for antibody delivery [13] and was included in this study as a reference cargo receptor system. TfR was moderately expressed in both hiPSC-BECs and Caco-2 cells and localized to both extra and intracellular surfaces (Fig. 2A, B). Sortilin was also moderately expressed in the two cell lines (Fig. 2A, B). Retrograde-transported sortilin was previously tested as an antibody cargo receptor and was found to transport antibodies to endolysosomal and trans-Golgi compartments with no recycling capacity [25]. The total stain in Fig. 2B suggests a similar localization in both hiPSC-BECs and Caco-2 cells. The cell surface localized receptors CD133 (Gene: *PROM1*) and podocalyxin (Gene: *PODXL*) are highly expressed in brain endothelial cells [33, 39, 43]. Total staining for these two receptors showed a highly polarized cell surface localization at the apical surface above the ZO1 stain (Fig. 2B–side view zx) with high expression of CD133 and podocalyxin in Caco-2 cells and hiPSC-BECs, respectively (Fig. 2A, B). Taken together, the two Transwell cell barrier setups enabled the study of two sorting receptors and two luminal cell surface receptors as cargo receptors for putative therapeutic antibodies.

### Intracellular antibody delivery by sorting receptors to early and late endosomes

**Fig. 3:**
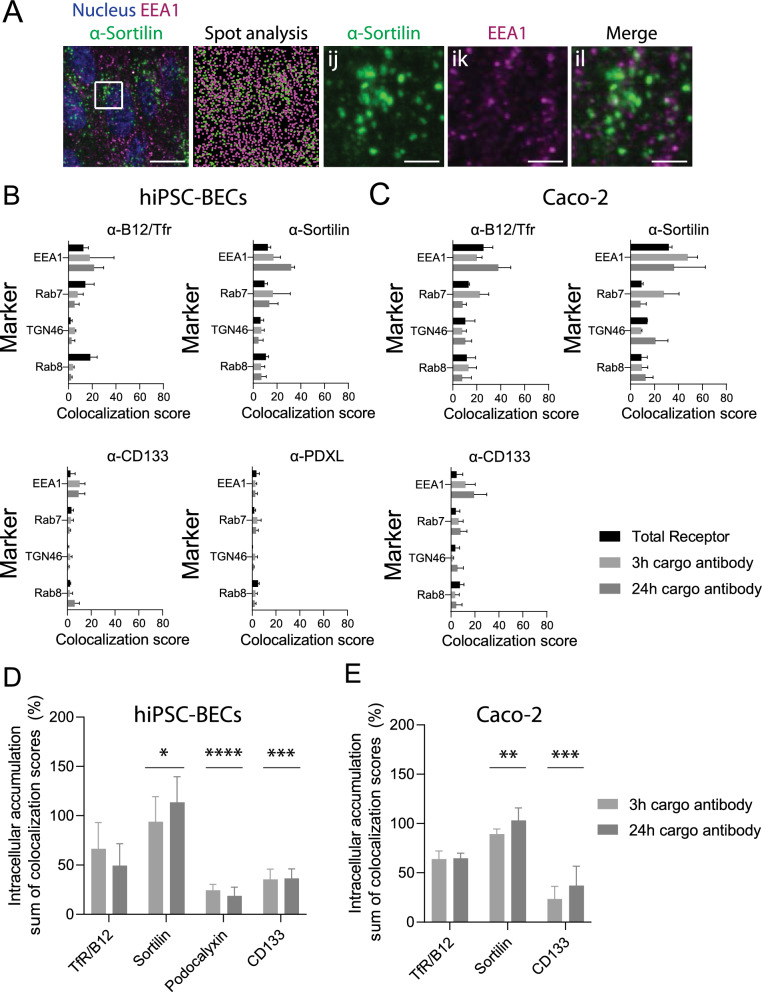
Internalization and subcellular localization of cargo antibodies by sorting and cell surface receptors. **A** Representative images (maximum projected z-stacks) showing immunofluorescence total staining of EEA1 (magenta) and 24 h cargo antibody against sortilin (green) in hiPSC-BECs with IMARIS spot analysis of the fluorescent signals. Scale bars are 10 µm. The white square inset is magnified in micrographs ij-il with scale bars showing 2 µm. **B**, **C** Colocalization analysis on hiPSC-BECs (**B**) and Caco-2 cells (**C**) of internalized cargo antibodies with selected markers in relation to the steady-state receptor (total receptor in black bars). See materials and methods for calculation of the colocalization score. See also Additional file 2: Fig S3 for the total spot measurements of receptor and cargo antibodies. **D**, **E** As an approximation for intracellular cargo antibody accumulation, the cargo antibodies colocalizing with the four intracellular markers were summed and analysed in relation to TfR cargo antibody accumulation. **D** Bar plots with sums for hiPSC-BECs and (**E**) for Caco-2 cells. Statistical significance was determined using two-way ANOVA with Tukey´s multiple comparisons test from three independent experiments. See also Additional file 2: Figs S3, S4

To study intracellular delivery and sorting, we applied imaging analysis with a partial sorting resolution in four of the major sorting compartments: the early/late sorting compartment marked by EEA1, the late endosomal compartment marked by Rab7, the trans-Golgi network marked by TGN46 and the secretory compartment (trans-Golgi–cell surface) marked by Rab8. Transport of cargo antibodies was initiated by adding 10 µg/ml (66.7 nM approximated from the standard weight ~ 150 kDa of IgG antibodies) cargo antibodies with three or 24 h of incubation to the apical chamber. Then, the cells were fixed, permeabilized and stained for cargo antibodies and intracellular markers. The fraction of antibody spots colocalizing with intracellular markers was quantified using a previously described method [11]. This approach enables analysis of an internalized fraction of cargo antibodies either in separate compartments, such as the early endosomal compartment (Fig. 3A and Additional file 2: Fig S4), or the overall distribution of total receptors in relation to their cargo antibodies (Fig. 3B, C). The quantification showed that TfR cargo antibodies were readily internalized into early (EEA1) and late (Rab7) endosomal compartments with little trafficking to the trans-Golgi (TGN46) or secretory compartments (Rab8), as shown for hiPSC-BECs in Fig. 3B and for Caco-2 cells in Fig. 3C. The same was evident for Sortilin, with an increased preference towards the late endosomal compartment (Fig. 3B, C). Strikingly, the cell surface receptors Podocalyxin and CD133 showed limited internalization of cargo antibodies in both cell models, as indicated by the low colocalization with intracellular compartments after 3 and 24 h of antibody incubation in Fig. 3B, C. This was not due to low receptor expression or a lack of cargo antibody binding since imaging and spot analysis showed high expression of the receptors and binding of cargo antibodies, respectively (Fig. 2 and Additional file 2: Fig S3C, D). Rather, imaging showed that a high degree of cargo antibody binding occurred at the cell surface, but the cargo antibody remained at the luminal cell surface even after 24 h (Additional file 2: Fig. S3A, B). To approximate the rate of internalized and accumulated cargo antibodies, colocalization scores were summed together in Fig. 3D, E and compared to the transport of TfR cargo antibodies. This showed that sortilin-directed cargo antibodies accumulate significantly more in the cell layer subcellular vesicles compared to TfR-directed cargo antibodies (Fig. 3D, E and Additional file 2: Fig. S3A, B). In contrast, luminal cell surface receptors supported significantly less intracellular delivery and accumulation of cargo antibodies (Fig. 3D, E and Additional file 2: Fig. S3A, B).

### Antibody transcytosis is restricted for sortilin and cell surface receptors

**Fig. 4:**
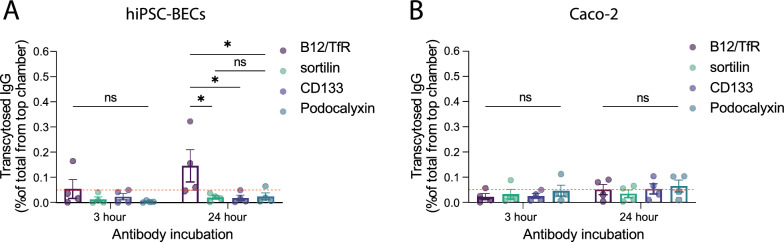
Transcytosis of cargo antibodies through cell barriers. **A** ELISA measurement of cargo antibody in the Transwell bottom acceptor chamber in relation to the added antibody in the top donor chamber given as transcytosed IgG percentage through the hiPSC-BEC layer. The red stippled line indicates the lower level of detection (approx. 50 pM) for the ELISA setup. **B** ELISA quantification of cargo antibody transcytosis through the Caco-2 cell layer. Statistical significance was determined using two-way ANOVA with Tukey´s multiple comparisons test based on four independent experiments. See also Additional file 2: Fig S5

TfR has previously proven effective for the delivery of bispecific therapeutic antibodies across the BBB. To measure the transcytosis capacity of antibodies through the experimental cell layers, a sandwich ELISA was employed with a lower limit of detection of ~ 50 pM (~ 7 ng/ml). This setup enabled the detection of transcytosed bispecific B12/TfR cargo antibody after incubating hiPSC-BECs for 24 h with antibody (Fig. 4A). Detection of the other cargo antibodies was not possible, suggesting no transcytosis of cargo antibodies via sortilin, podocalyxin or CD133 receptors. Surprisingly, no transcytosed B12/TfR antibody was measured transcytosing through the Caco-2 cell layer, suggesting different intracellular sorting in the two cell lines. Cargo antibodies directed against sortilin showed the significantly largest capacity for intracellular delivery (Fig. 3) but with no detectable transcytosis (Fig. 4). Similar experimentation using the hiPSC-BECs without prior NCC with astrocytes did not change these transport properties (Additional file 2: Fig S5).

### Therapeutic delivery to the BMEC cell layer can be mediated via sortilin

**Fig. 5:**
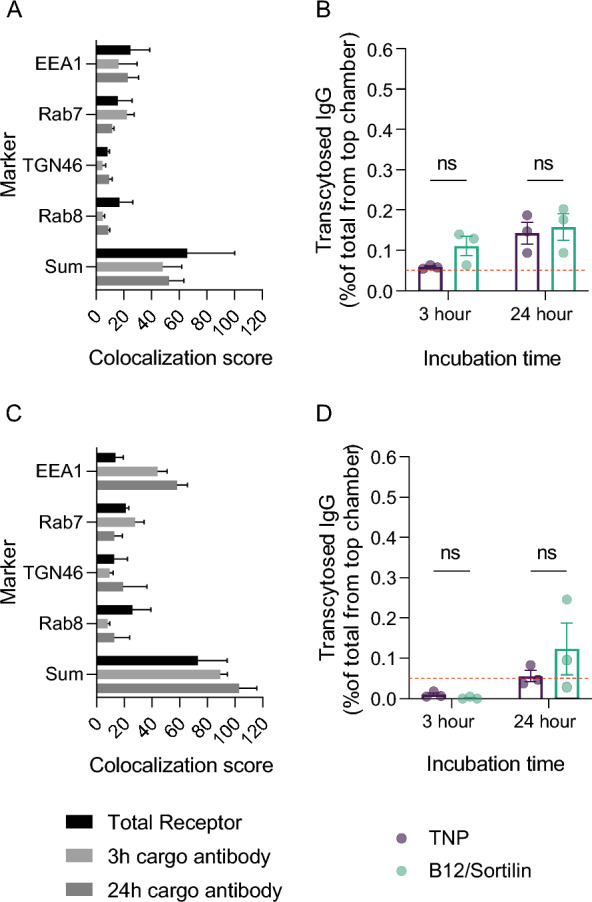
Bispecific cargo antibody hitchhiking on sortilin shows intracellular accumulation. Bar plots show colocalization scores with sums (**A**) and transcytosis (**B**) analysis using 10 µg/ml bispecific human anti-B12/sortilin construct on hiPSC-BECs. The bar plot in (**C**, **D**) shows the equivalent analysis for Caco-2 cells. A humanized control antibody targeting trinitrophenol (TNP, not expressed by mammals) was used as a parallel control (**B**, **E**). TNP was also tested by immunofluorescence staining, which showed no binding or internalization into the examined cell layers. Statistical significance was determined using ordinary two-way ANOVA test based on three independent experiments

To address whether sortilin could work as a functional receptor for the delivery of therapeutic antibodies into the cell layer itself, a bispecific B12/sortilin antibody variant was generated similar to the anti-B12/TfR construct. The antibody showed intracellular accumulation and no detectable transcytosis capacity (Fig. 5A, B) in hiPSC-BECs but had increased transcytosis capacity through the Caco-2 cell layer (Fig. 5C, D).

## Discussion

Cellular receptors form antennas to mediate signalling, transfer cargo or anchor molecules at cell membranes. A subset of receptors are expressed in human brain capillary endothelial cells and could work as putative cargo receptors for therapeutic delivery. Alzheimer’s disease is one of the major neuronal diseases and is targeted by therapeutics such as antibodies capable of crossing the BBB. For this purpose, putative antibody cargo receptors should also be expressed in pathological situations. In this study, immunohistochemical staining of tissue sections spanning all cortical layers of the grey matter of the superior occipital gyrus from human control and Alzheimer’s disease cases showed no downregulation of selected cargo receptors. Thus, we validated the expression of the selected receptors as putative cargo receptors, e.g., for Alzheimer’s disease. One of the selected receptors, TfR, functions to maintain receptor-mediated transport (RMT) of iron via transferrin into the cell. High-affinity cargo antibodies derail the TfR receptor from recycling into the degradative compartment [2], hence, the use of TfR as a cargo receptor for the delivery of therapeutic antibodies might affect its sorting. In turn, this can have profound effects on physiology [28] and the ability to maintain a healthy brain [23]. An optimal scenario in drug delivery to the brain would be to develop a therapeutic antibody construct where a therapeutic entity could hitchhike via a cargo receptor for its functional destination without perturbing the sorting and functional ability of the cargo receptor. Increasing numbers of reports have described the use of hiPSC-BECs as an exploratory research model of the human BBB to study therapeutic delivery [7, 27, 29]. To study the functionality of the putative cargo receptor types, we also used a Transwell BBB model with a hiPSC-BEC layer. Here, we chose a human iPS cell line named SBAD-02-01 with no restrictions for commercial research. With respect to barrier properties, hereunder polarised transporter activity (personal communication with Birger Brodin, Copenhagen University), tightness and expression of endothelial markers (claudin5, glut1, occludin and VE-cadherin) the SBAD-02-01 cells performs as other human iPS cell lines [8, 29, 34], thereby generating a tight barrier with BBB properties, which recapitulates the ability to sort a bispecific B12/TfR antibody construct for transcytosis delivery.

Although TfR has become the favourite cargo receptor for transcytosis across the BBB, a recent study showed bidirectional trafficking and endocytic capacity at both luminal and abluminal surfaces in BECs [26]. The study further shows transcytosis of cargo antibodies in both directions, thus limiting focused transcytosis in one direction [26]. The recycling capacity of TfR is a major determinant of its function in transporting transferrin. In polarized MDCK cells [31], the recycling functionality of a receptor is a feature that promotes sorting for transcytosis. Similarly, our data suggests that the recycling capacity of a receptor could be an indicator of its transcytosis capacity in hiPSC-BECs and possibly BECs in general. With this notion, a large receptor family, the G-protein coupled receptor family, is known to be constitutively recycled [22]. Due to this feature of the G-protein coupled receptor family, it should be considered whether this receptor family could harbour putative cargo receptors expressed in BECs, which could be harnessed for therapeutic delivery to the brain.

Much effort has been put into identifying novel brain capillary receptors with potential as cargo receptors for therapeutic delivery to the brain [12, 47, 49]. One proposed receptor is podocalyxin, a luminal cell surface receptor that was found to readily internalize in the immortalized human brain and nonpolarized capillary endothelial cell line hCMEC/D3 [12]. Here, we show that podocalyxin and another luminal surface-positioned receptor, CD133, exhibit static positioning in polarized cell layers with a poor capacity to internalize cargo antibodies. How cell surface proteins are sorted to the luminal surface and kept in that position is not well understood, but encrypted motifs and polarized sorting machinery probably set the stage for regulating the luminal positioning of proteins [41]. These features and missing intracellular sorting motifs might inhibit effective internalization of cargo antibodies into the cell layer via the luminal cell-surface type of receptor. Hence, proteins that are prone to traffic between the cell surface and intracellular sorting compartments are better-suited candidates to explore as cargo receptors for therapeutic constructs. However, it should not be excluded that these kinds of luminal cell surface localized receptors could be used for docking entities to the luminal brain endothelium surface.

Sorting receptors harbouring a VPS10P domain form a promiscuous family of signalling and cargo receptors with many cargoes and functionalities important for neuronal health [21]. Within this receptor family, the sortilin receptor is a particularly dynamic receptor that traffics between the cell surface, endosomes and the trans-Golgi network [25]. The receptor is expressed in the BBB [36] and readily internalizes antibodies without the capacity to recycle [25]. This feature might serve well for delivering therapeutic constructs for the endothelial cell layer itself. In this study, we found sortilin to be superior to TfR for the internalization of antibodies into the cell layer but with less or no capacity for transcytosis. Although we observed a high intracellular accumulation of Sortilin cargo antibodies compared to TfR cargo antibodies, we surprisingly did not observe a dominant accumulation of Sortilin cargo antibodies in late endosomes (Rab7) or the TGN, as expected from previous publications [25]. However, a recently published article by van der Beek et al. interestingly demonstrated that EEA1 is also partly a marker for the early part of late endosomes [38]. These subtle but determinant sorting effects highlight the need for better mapping of the endo-lysosomal system to understand the sorting and transcytosis mechanisms in BECs. Delivery of therapeutics to BECs [17] is becoming a warranted strategy to alleviate brain disorders caused by dysregulation in the cerebrovascular network [35]. *SORT1,* encoding sortilin, is a cardiovascular risk gene [14], therefore, usage of the receptor as a cargo receptor needs consideration to avoid derailing its functions. Given the recent advantages in therapeutic antibody design [48], how low-affinity sortilin-directed cargo antibodies deliver therapeutic moieties to the BEC layer could be explored.

Here, we benchmarked two types of receptors for their capacity to transport cargo antibodies into a barrier cell layer and for their ability to sort for transcytosis. Due to the debated epithelial properties of the human BBB model established with hiPSC-BECs [20], we compared all analyses made with the hiPSC-BECs to a similar barrier setup with epithelial Caco-2 cells, as Caco-2 cells are still used as a barrier system to model BBB properties. This comparison showed similar properties for the investigated receptor types, except for antibody sorting via TfR. Here, antibody transcytosis occurred through the hiPSC-BEC layer but was absent in the Caco-2 cell layer. TfR-mediated delivery of therapeutic antibodies has been shown in the nonhuman primate brain, suggesting that hiPSC-BECs are a better model for recapitulating BBB properties than Caco-2 cells.

The ELISA assay for this study took advantage of capture antibodies specific for the FC region of IgG. This setup allowed for detecting of cargo antibody in the receiver chamber at 50 pico molar concentrations. We found that the media formulation used for the cell setups were a limitation to the sensitivity but were unable to change these in order to enable coherence and reproducibility with previous and coming studies. Interestingly, a study from the Hultqvist group [24] suggests the possibility to lower the detection level of cargo IgGs using IgG F(ab´)_2_–specific antibodies as capture antibodies. Future experimentation will show if this will enable bypass of the medium background used for the reported cell systems to increase the detection limit and broaden the biological transcytosis readout.

Based on our data, we highlight sorting receptors as cargo receptors for intracellular delivery to the barrier cell layer and find that the recycling functionality of sorting receptors is the most important feature for sorting antibodies for transcytosis. The highly expressed BBB cell surface receptors podocalyxin and CD133 are poor cargo receptors for antibody delivery despite their availability on the luminal surface of cells, which may be explained by low mobility between compartments in polarized cell layers. We propose sortilin as a putative cargo receptor for delivering therapeutic antibodies to alleviate the diseased BBB endothelial cell layer.

### Supplementary Information

**Additional file 1.**

**Additional file 2: Figure S1.** Verification of cargo antibody specificities. (A-D) Western blotting analysis of antibody specificity indicated by single bands in human and mouse lysates (6 μg loaded per lane). (E) The diagram illustrates how parent bivalent K409R or F405L mutated antibodies were resembled into bispecific monovalent antibodies for this study using controlled fab-arm exchange. (F-I) Immunofluorescence total staining of hiPSC-BECs. Humanized bispecific antibody construct (Genentech 15G11 origin) targeting HIV G120 (B12) and TfR shows single band detection in human cell lysates (A) and binding to SBAD-02-01 expressed TfR (F). Both mouse anti-sortilin (B and G) and mouse anti-podocalyxin (C and H) show specificity to their human antigens with cross-species detection towards mouse variants in mouse capillary lysates (B and C). Mouse anti-CD133 shows specific detection of human CD133 (D and I) with no detection of mouse variants in bEnd.3 or mouse capillary lysates (D). Scale bars are 10 μm.** Figure S2.** Effect of medium change on barrier tightness for transport experiments. (A-B) Total receptor stains showing receptor localization after changing medium into transport medium. Cells are stained for ZO1 in white and the indicated receptors in green. (C-D) TEER measurements before medium change (Start), three hours after medium change (three-hour time-point for addition of cargo antibody spike solution) and 24 hours (24 hours after cargo antibody spike time point). TEER values were measured using an EVOM Epithelial Volt/Ohm meter (World Precision Instruments, Friedberg, Germany) with chopstick electrodes. The TEER measurement of a cell-free collagen IV/fibronectin-coated filter was used as blank, which was subtracted from TEER values measured on filter-seeded cells. Bar plots show mean values ±SD based on three independent experiments. (F) Sodium fluorescein (NaF) was used to measure paracellular permeability through the hiPSC-BEC layer. The box plot show mean value ±SD of three independent experiments. (F) Representative micrographs of four selected endothelial markers stained with antibodies described before (Hudecz et al., 2023). See also supplementary table 1 for RNA expression analysis. Scale bars are 10 μm.** Figure S3.** Positioning of cargo antibodies in cell layers. (A-B) Micrographs show side views (x-z) of maximum projected z-stacks. Cells are stained for ZO1 in white and the indicated receptors in green. Sorting receptors TfR and sortilin are both expressed in hiPSC-BECs and Caco-2 cells and are localized at intracellular surfaces. The polarized receptors podocalyxin and CD133 are both expressed in the apical surface facing the luminal side of the Transwell chamber. The dashed blue lines in (A-B) indicates the filter surface (abluminal cell surface). Scale bars are 5 μm. (C-D) Receptor expression (total stain) and cargo antibody signals were analysed using immunofluorescent spot analysis for the indicated receptors in hiPSC-BECs (C) and similarly in for Caco2 cells in (D). All bar plots show Mean ±SD based on three independent experiments.** Figure S4.** Representative control immunostainings for markers and cargo receptors. (A) Representative maximum projected z-stack micrographs from stains against selected markers of the study. (B-C) Maximum projected z-stack of micrographs from total receptor stains showing receptor localization after changing medium into transport medium. The x-y areas marked by the white stippled line are shown as x-z side views. Scale bars are 10 μm.** Figure S5.** Sorting and transcytosis of cargo antibodies through hiPSC-BECs without astrocyte coculture. (A) Colocalization analysis of internalized cargo antibodies with selected markers in relation to the steady-state receptor (total receptor in black bars). (B) ELISA measurement of antibody in the Transwell bottom acceptor chamber in relation to the added antibody in the top donor chamber given as transcytosed IgG percentage through the hiPSC-BEC layer (without prior astrocyte NCC). The red stippled line indicates the lower level of detection (approx. 50 pM) for the ELISA setup. Statistical significance was determined using two-way ANOVA with Tukey´s multiple comparisons test. All bar plots show Mean ±SD based on three or four independent experiments.

## Data Availability

All data and materials are available upon reasonable request.
