# Supplementary material for: Subcellular trafficking and transcytosis efficacy of different receptor types for therapeutic antibody delivery at the blood‒brain barrier
Source: Fluids Barriers CNS. 2023 Nov 6;20:82. doi: 10.1186/s12987-023-00480-x (PMC10626680; doi:10.1186/s12987-023-00480-x)
Supplement: Supplementary file 2 — Additional file 2: Figure S1. Verification of cargo antibody specificities. (A-D) Western blotting analysis of antibody specificity indicated by single bands in human and mouse lysates (6 μg loaded per lane). (E) The diagram illustrates how parent bivalent K409R or F405L mutated antibodies were resembled into bispecific monovalent antibodies for this study using controlled fab-arm exchange. (F-I) Immunofluorescence total staining of hiPSC-BECs. Humanized bispecific antibody construct (Genentech 15G11 origin) targeting HIV G120 (B12) and TfR shows single band detection in human cell lysates (A) and binding to SBAD-02-01 expressed TfR (F). Both mouse anti-sortilin (B and G) and mouse anti-podocalyxin (C and H) show specificity to their human antigens with cross-species detection towards mouse variants in mouse capillary lysates (B and C). Mouse anti-CD133 shows specific detection of human CD133 (D and I) with no detection of mouse variants in bEnd.3 or mouse capillary lysates (D). Scale bars are 10 μm. Figure S2. Effect of medium change on barrier tightness for transport experiments. (A-B) Total receptor stains showing receptor localization after changing medium into transport medium. Cells are stained for ZO1 in white and the indicated receptors in green. (C-D) TEER measurements before medium change (Start), three hours after medium change (three-hour time-point for addition of cargo antibody spike solution) and 24 hours (24 hours after cargo antibody spike time point). TEER values were measured using an EVOM Epithelial Volt/Ohm meter (World Precision Instruments, Friedberg, Germany) with chopstick electrodes. The TEER measurement of a cell-free collagen IV/fibronectin-coated filter was used as blank, which was subtracted from TEER values measured on filter-seeded cells. Bar plots show mean values ±SD based on three independent experiments. (F) Sodium fluorescein (NaF) was used to measure paracellular permeability through the hiPSC-BEC layer. The box plot show mean v [file 12987_2023_480_MOESM2_ESM.pdf]

Additional file

Subcellular trafficking and transcytosis efficacy of different receptor types for therapeutic antibody delivery at the blood–brain barrier

Mikkel Roland Holst[1], Nienke M. de Wit[2,3], Burak Ozgür[4,5], Andreas Brachner[6], Kathrine Hyldig[1, 5], Antje Appelt-Menzel[7,8], Hannah Sleven[9], Zameel Cader[9], Helga E. de Vries[2,3], Winfried Neuhaus[6,10], Allan Jensen[5], Birger Brodin[4] and Morten Schallburg Nielsen[1]*

This document includes:

Supplementary figures with figure legends

Supplementary material and methods

Supplementary references

Additional figures

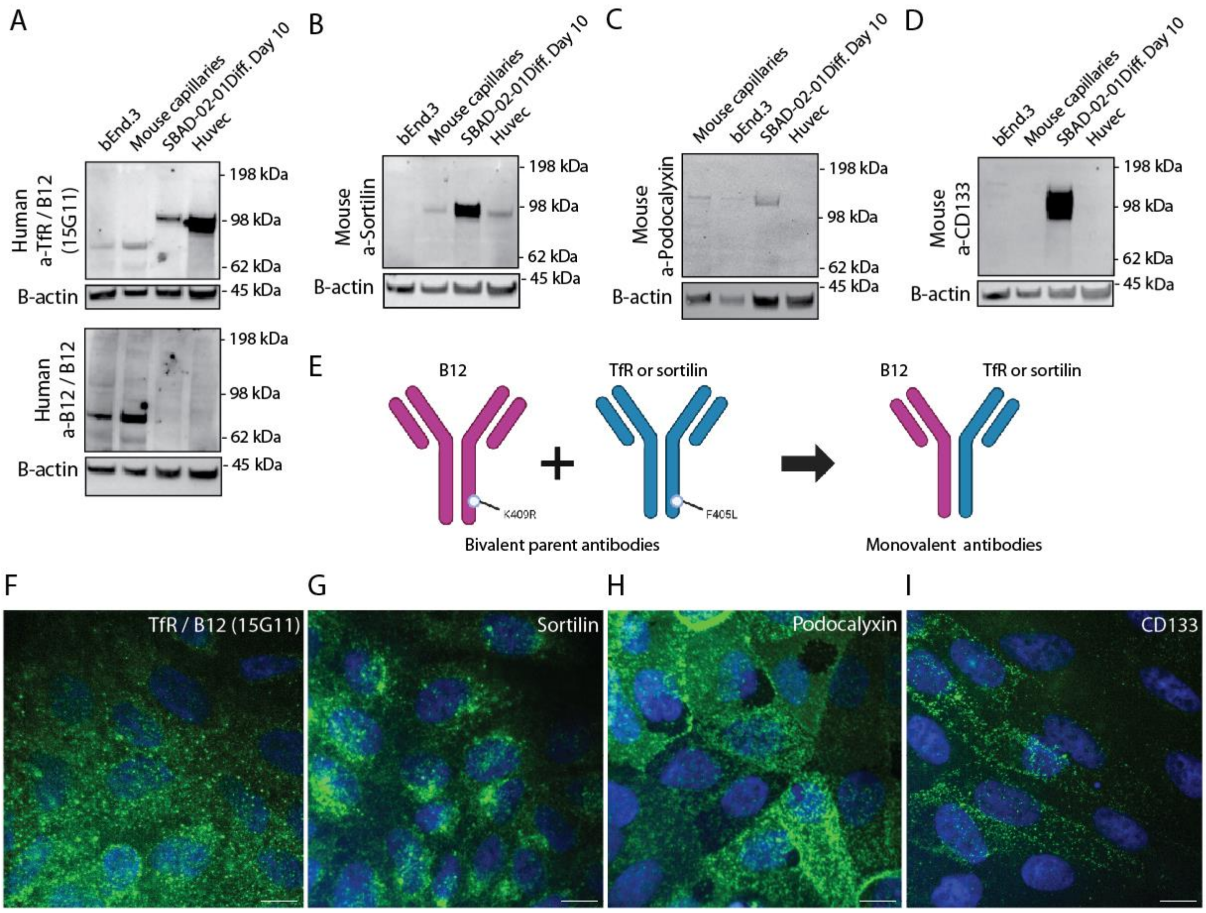

Figure S1. Verification of cargo antibody specificities. (A-D) Western blotting analysis of antibody specificity indicated by single bands in human and mouse lysates (6 µg loaded per lane). (E) The diagram illustrates how parent bivalent K409R or F405L mutated antibodies were resembled into bispecific monovalent antibodies for this study using controlled fab-arm exchange. (F-I) Immunofluorescence total staining of hiPSC-BECs. Humanized bispecific antibody construct (Genentech 15G11 origin) targeting HIV G120 (B12) and TfR shows single band detection in human cell lysates (A) and binding to SBAD-02-01 expressed TfR (F). Both mouse anti-sortilin (B and G) and mouse anti-podocalyxin (C and H) show specificity to their human antigens with cross-species detection towards mouse variants in mouse capillary lysates (B and C). Mouse anti-CD133 shows specific detection of human CD133 (D and I) with no detection of mouse variants in bEnd.3 or mouse capillary lysates (D). Scale bars are 10 µm.

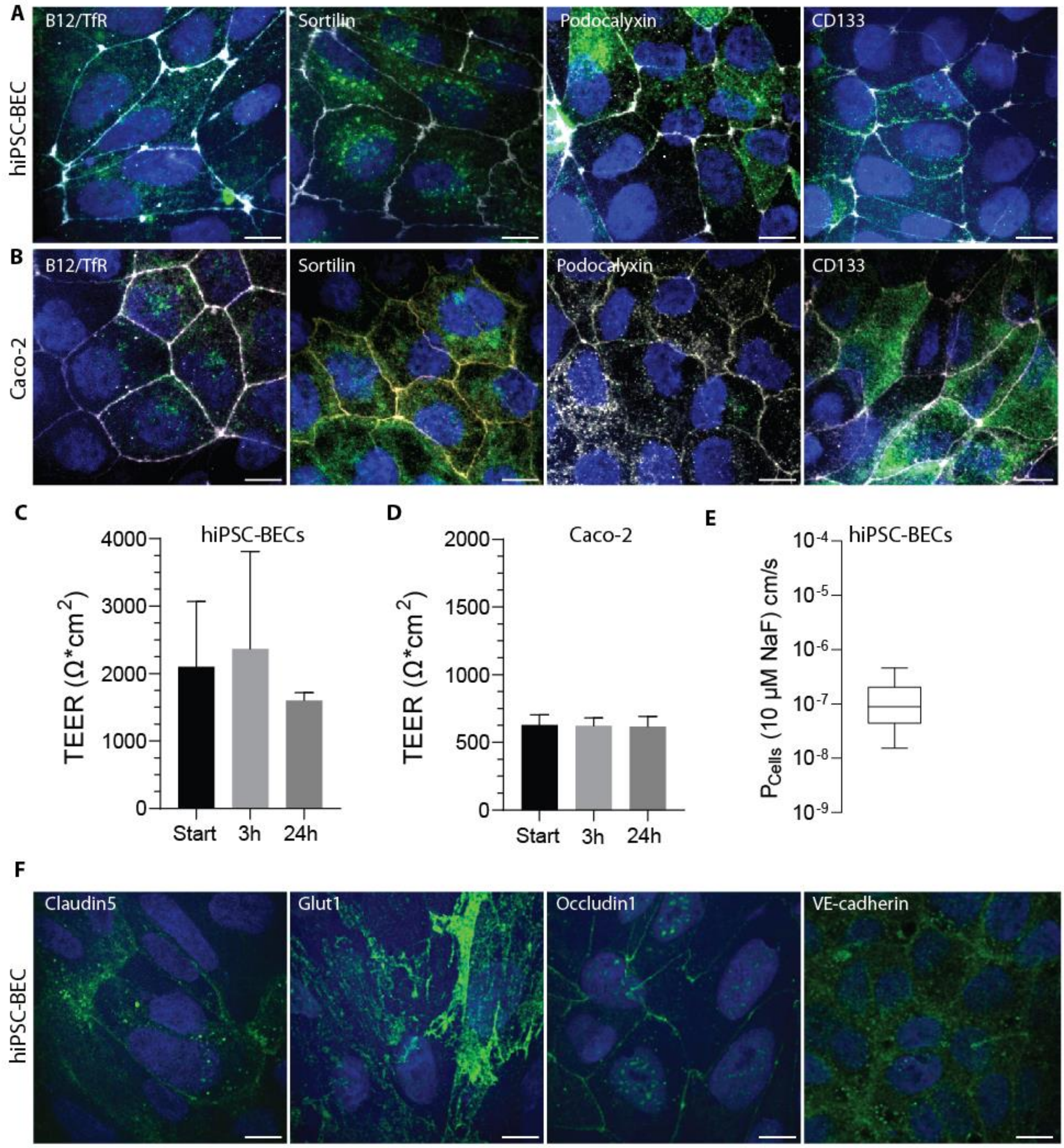

Figure S2. Effect of medium change on barrier tightness for transport experiments. (A-B) Total receptor stains showing receptor localization after changing medium into transport medium. Cells are stained for ZO1 in white and the indicated receptors in green. (C-D) TEER measurements before medium change (Start), three hours after medium change (three-hour time-point for addition of cargo antibody spike solution) and 24 hours (24 hours after cargo antibody spike time point). TEER values were measured using an EVOM Epithelial Volt/Ohm meter (World Precision Instruments, Friedberg, Germany) with chopstick electrodes. The TEER measurement of a cell-free collagen IV/fibronectin-coated filter was used as blank,

which was subtracted from TEER values measured on filter-seeded cells. Bar plots show mean values ±SD based on three independent experiments. (F) Sodium fluorescein (NaF) was used to measure paracellular permeability through the hiPSC-BEC layer. The box plot show mean value ±SD of three independent experiments. (F) Representative micrographs of four selected endothelial markers stained with antibodies described before (Hudecz et al., 2023). See also supplementary table 1 for RNA expression analysis. Scale bars are 10 µm.

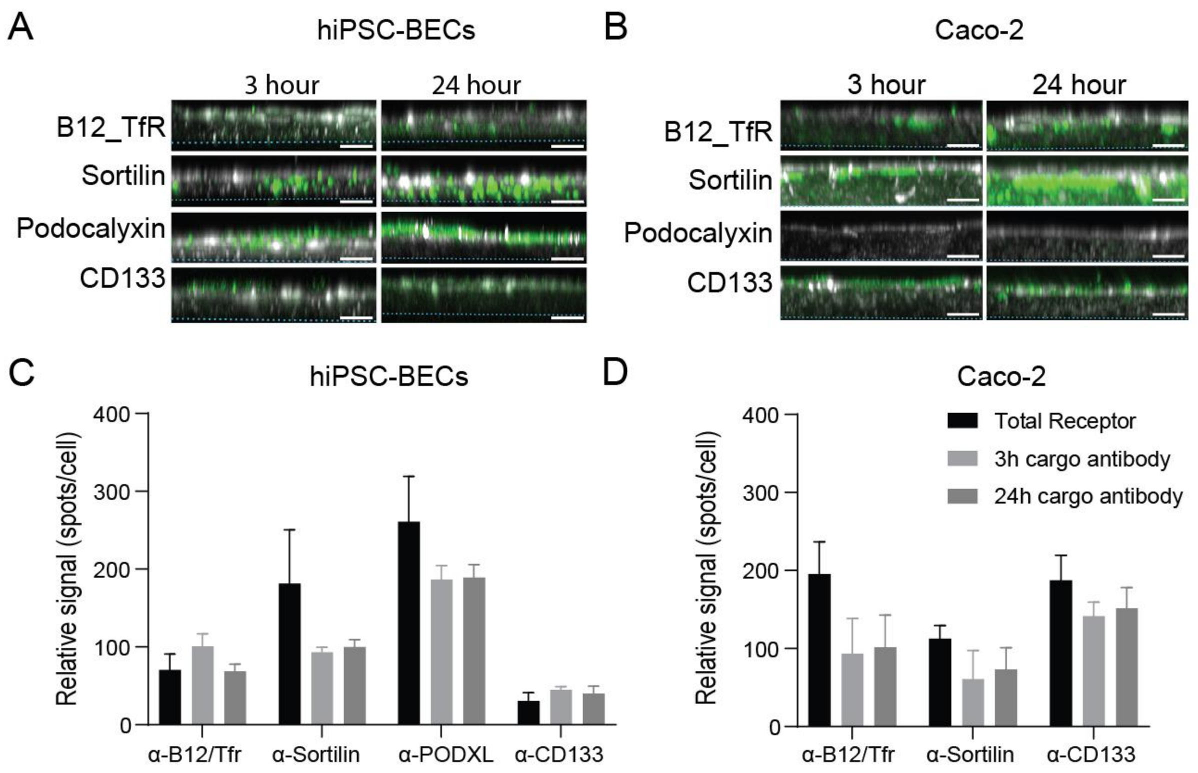

Figure S3. Positioning of cargo antibodies in cell layers. (A-B) Micrographs show side views (x-z) of maximum projected z-stacks. Cells are stained for ZO1 in white and the indicated receptors in green. Sorting receptors TfR and sortilin are both expressed in hiPSC-BECs and Caco-2 cells and are localized at intracellular surfaces. The polarized receptors podocalyxin and CD133 are both expressed in the apical surface facing the luminal side of the Transwell chamber. The dashed blue lines in (A-B) indicates the filter surface (abluminal cell surface). Scale bars are 5 µm. (C-D) Receptor expression (total stain) and cargo antibody signals were analysed using immunofluorescent spot analysis for the indicated receptors in hiPSC-BECs (C) and similarly in for Caco2 cells in (D). All bar plots show Mean ±SD based on three independent experiments.

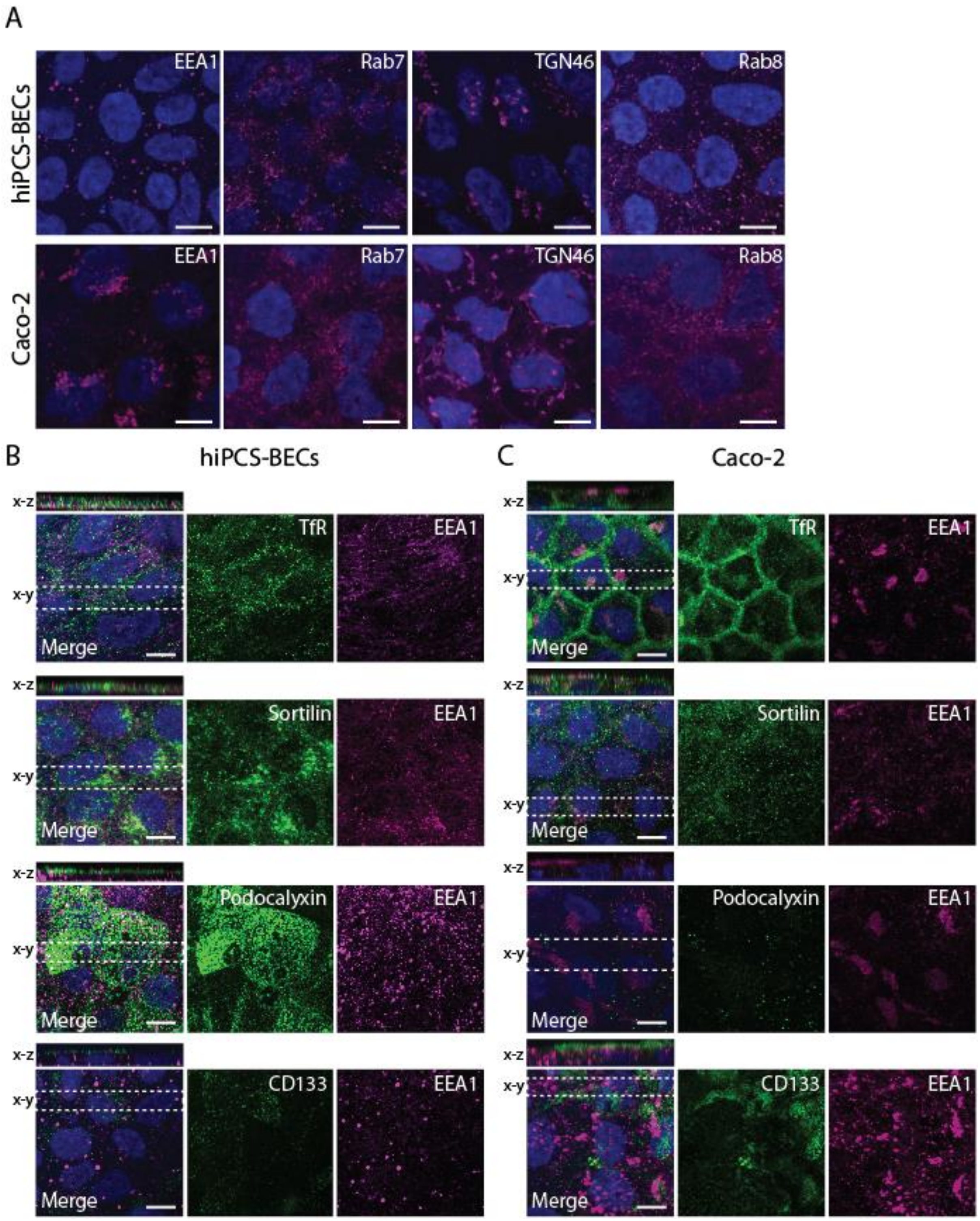

Figure S4. Representative control immunostainings for markers and cargo receptors. (A) Representative maximum projected z-stack micrographs from stains against selected markers of the study. (B-C) Maximum projected z-stack of micrographs from total receptor stains showing receptor localization after changing medium into transport medium. The x-y areas marked by the white stippled line are shown as x-z side views. Scale bars are 10 µm.

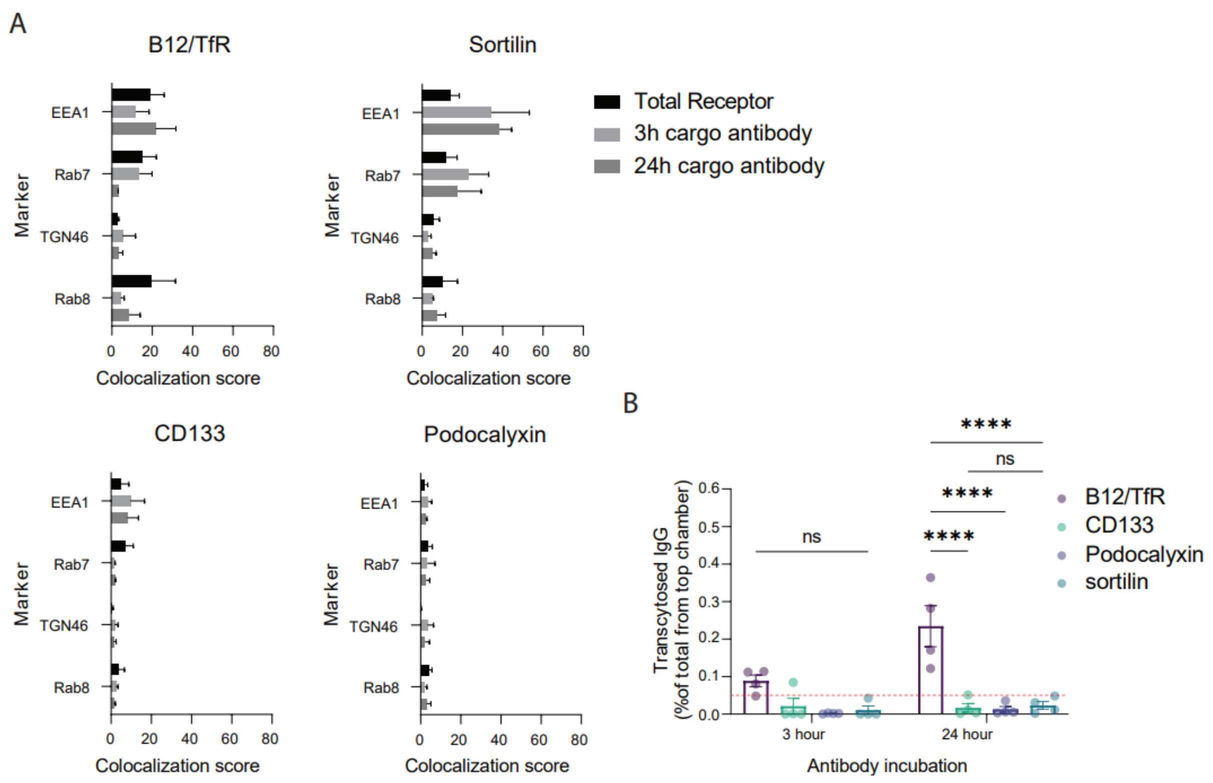

Figure S5. Sorting and transcytosis of cargo antibodies through hiPSC-BECs without astrocyte coculture. (A) Colocalization analysis of internalized cargo antibodies with selected markers in relation to the steady-state receptor (total receptor in black bars). (B) ELISA measurement of antibody in the Transwell bottom acceptor chamber in relation to the added antibody in the top donor chamber given as transcytosed IgG percentage through the hiPSC-BEC layer (without prior astrocyte NCC). The red stippled line indicates the lower level of detection (approx. 50 pM) for the ELISA setup. Statistical significance was determined using two-way ANOVA with Tukey´s multiple comparisons test. All bar plots show Mean ±SD based on three or four independent experiments.

Materials and Methods

High-throughput qPCR

Analysis was performed as previously published (Daullary et al., 2023; Gerhartl et al., 2020; Koenig et al., 2022; Krasemann et al., 2022; Lin et al., 2020; Ramme et al., 2019; Singh et al., 2023). RNA was harvested and isolated according to the manufacturer's instructions using the RNAeasy® Plus mini Kit (Qiagen, Cat. no.: 7434). A total volume of 20 µl cDNA was synthesized from 250 ng RNA using the High Capacity cDNA Reverse Transcriptase Kit (Thermo Fisher Scientific, cat. no.: 4368814). After preamplification of the targets in the samples, the high-throughput qPCR chip was performed with the preamplified cDNA in 96x96 chips applying the Biomark™ system (Fluidigm®) for following targets: *ABCA1, ABCA7, ABCB1, ABCC1, ABCC2, ABCC3, ABCC4, ABCC5 tv1+tv3, ABCG2, AGER, APOE, AQP3, AQP5, AQP10, AQP11, CDH1, CDH5 CLDN1, CLDN2 tv3, CLDN3, CLDN4, CLDN5, CLDN6, CLDN7, CLDN8, CLDN9, CLDN10 tva, CLDN10 tvb, CLDN11, CLDN12 tv1, CLDN12 tv2, CLDN12 tv3, CLDN14, CLDN15, CLDN16, CLDN17, CLDN18 tv1b, CLDN18 tv2a, CLDN19 tv1, CLDN20, CLDN21, CLDN22, CLDN23, CLDN24, CLDN25, CLDN26 tv3, CLDN27, CTNNB tv1-3,_F11R, FN1, INSR, JAM2, JAM3, KRT8, KRT18, KRT19, LRP1, LRP8, LSR, MARVELD2, MFSD2A, MUC1 tva, MUC1 tvb, MUC18, MUC20, OCLN, PECAM1, RARA tv1-4, RARB tva, RARB tvb, RXRA tv1, RXRB tv2, S100A4 tv1, S100A4 tv2, SLC2A1, SLC7A1, SLC7A3, SLC7A5, SLC16A1, SLC16A2, SLC29A1, TFRC, TJP1, TJP2, TJP3 tv2, VEGFA, VIM, VWF, WWC2.* Threshold cycle (Ct) values were normalized to the geometric mean of endogenous housekeeping genes *PPIA, ACTB, GAPDH* and *B2M,* and relative quantification was performed based on $2^{-\Delta Ct}$ method.

Paracellular Permeability of NaF

For measurement of paracellular permeability, 500,000 day 8 differentiated (D8) SBAD-02-01 cells were seeded per 12-well filter insert (GREINER BIO-ONE, Cat. No.: GR-665641). Cells were differentiated for experimental day 10 (D10). On D10 Transwell chambers were replenished with 700 µl (apical chamber) and 1500 µl (basolateral chamber) using EC medium consisting of human endothelial serum-free medium (Gibco, cat. No.: 11111044) supplemented with 0.5% B-27™ supplement (50X) (Gibco, cat. No.: 17504001). A spike stock with 200 µM sodium fluorescein

(NaF, Sigma-Aldrich #F6377-100G) was prepared in EC medium. The transcellular transport experiment was initiated by removing 35 µl medium from the apical chamber and adding back 35 µl 200 µM NaF spike stock for a final concentration of 10 µM NaF in the apical chamber. Empty filters were similarly added NaF for extracting the effect of the filter. Transwells with cells were placed on an orbital shaker at 100 rpm rotation in a 37 °C incubator. Samples were collected from apical and basolateral chambers after 60 minutes and transferred to a 96-well plate. The fluorescence intensity of the sample was measured by a CLARIOstar® Plus microplate reader (BMG Labtech, Ortenberg, Germany) (λ_{Exc} = 485 nm, λ_{Em} = 515 nm, Bandwidth = 15 and 20 nm, respectively). Relative fluorescence units (RFU) were measured within the linear range of the spectrophotometer (tested by a regression test) and the slopes were calculated to extrapolate the apparent permeability coefficients (P_{app}) of transcellular transport based on the clearance principle (Siflinger-Birnboim et al., 1987).

Calculation of transcellular permeability P_{cells}

First, the apparent permeablities was calculated for both filters with cells and filters alone using the measured RFU units:

$$P_{app} = \frac{RFU_{basolateral\ chamber}}{RFU_{apical\ chamber} \cdot A(cm2) \cdot t(sec)}$$

Where $RFU_{basolateral\ chamber}$ represent the receiver compartment at time = 60 minutes $RFU_{apical\ chamber}$ is the initial donor compartment concentration in the apical chamber at t=0, A is the area of the filter (1.13 cm^2) and t the time (3600 sec).

The final cellular permeability (P_{cells}) was calculated by extracting the filter effect:

$$\frac{1}{P_{cells}} = \frac{1}{P_{app,hiPSC-BECs+filter}} - \frac{1}{P_{app,filter}}$$

Where $P_{app,hiPSC-BECs+filter}$ is the apparent permeability of the cell monolayer with filter, and $P_{app,filter}$ is the apparent permeability of the collagen IV/fibronectin-coated blank filter.

References

Daullary, T., Imdahl, F., Dietrich, O., Hepp, L., Krammer, T., Fey, C., Neuhaus, W., Metzger, M., Vogel, J., Westermann, A.J., *et al.* (2023). A primary cell-based in vitro model of the human small intestine reveals host olfactomedin 4 induction in response to Salmonella Typhimurium infection. Gut Microbes *15*, 2186109.

Gerhartl, A., Pracser, N., Vladetic, A., Hendrikx, S., Friedl, H.P., and Neuhaus, W. (2020). The pivotal role of micro-environmental cells in a human blood-brain barrier in vitro model of cerebral ischemia: functional and transcriptomic analysis. Fluids Barriers CNS *17*, 19.

Hudecz, D., McCloskey, M.C., Vergo, S., Christensen, S., McGrath, J.L., and Nielsen, M.S. (2023). Modelling a Human Blood-Brain Barrier Co-Culture Using an Ultrathin Silicon Nitride Membrane-Based Microfluidic Device. Int J Mol Sci *24*.

Koenig, L., Ramme, A.P., Faust, D., Mayer, M., Flotke, T., Gerhartl, A., Brachner, A., Neuhaus, W., Appelt-Menzel, A., Metzger, M., *et al.* (2022). A Human Stem Cell-Derived Brain-Liver Chip for Assessing Blood-Brain-Barrier Permeation of Pharmaceutical Drugs. Cells *11*.

Krasemann, S., Haferkamp, U., Pfefferle, S., Woo, M.S., Heinrich, F., Schweizer, M., Appelt-Menzel, A., Cubukova, A., Barenberg, J., Leu, J., *et al.* (2022). The blood-brain barrier is dysregulated in COVID-19 and serves as a CNS entry route for SARS-CoV-2. Stem Cell Reports *17*, 307-320.

Lin, G.C., Leitgeb, T., Vladetic, A., Friedl, H.P., Rhodes, N., Rossi, A., Roblegg, E., and Neuhaus, W. (2020). Optimization of an oral mucosa in vitro model based on cell line TR146. Tissue Barriers *8*, 1748459.

Ramme, A.P., Koenig, L., Hasenberg, T., Schwenk, C., Magauer, C., Faust, D., Lorenz, A.K., Krebs, A.C., Drewell, C., Schirrmann, K., *et al.* (2019). Autologous induced pluripotent stem cell-derived four-organ-chip. Future Sci OA *5*, FSO413.

Siflinger-Birnboim, A., Del Vecchio, P.J., Cooper, J.A., Blumenstock, F.A., Shepard, J.M., and Malik, A.B. (1987). Molecular sieving characteristics of the cultured endothelial monolayer. J Cell Physiol *132*, 111-117.

Singh, N.R., Gromnicova, R., Brachner, A., Kraev, I., Romero, I.A., Neuhaus, W., and Male, D. (2023). A hydrogel model of the human blood-brain barrier using differentiated stem cells. PLoS One *18*, e0283954.
